# Isolation and Crystallization of the D156C Form of Optogenetic ChR2

**DOI:** 10.3390/cells11050895

**Published:** 2022-03-05

**Authors:** Liying Zhang, Kaituo Wang, Shuo Ning, Per Amstrup Pedersen, Annette Susanne Duelli, Pontus Emanuel Gourdon

**Affiliations:** 1 Department of Biomedical Sciences, University of Copenhagen, Nørre Allé 14, DK-2200 Copenhagen, Denmark; liying.zhang@sund.ku.dk (L.Z.); kaituo@sund.ku.dk (K.W.); annette.duelli@gmail.com (A.S.D.); 2 Key Laboratory of Molecular Medicine and Biotherapy, School of Life Science, Beijing Institute of Technology, Beijing 100081, China; 3120195701@bit.edu.cn; 3 Department of Biology, University of Copenhagen, Universitetsparken 13, DK-2100 Copenhagen, Denmark; papedersen@bio.ku.dk; 4 Department of Experimental Medical Science, Lund University, Sölvegatan 19, SE-221 84 Lund, Sweden

**Keywords:** optogenetics, channelrhodopsin-2, open state, production, purification, crystallization

## Abstract

Channelrhodopsins (ChRs) are light-gated ion channels that are receiving increasing attention as optogenetic tools. Despite extensive efforts to gain understanding of how these channels function, the molecular events linking light absorption of the retinal cofactor to channel opening remain elusive. While dark-state structures of ChR2 or chimeric proteins have demonstrated the architecture of non-conducting states, there is a need for open- and desensitized-state structures to uncover the mechanistic principles underlying channel activity. To facilitate comprehensive structural studies of ChR2 in non-closed states, we report a production and purification procedure of the D156C form of ChR2, which displays prolonged channel opening compared to the wild type. We demonstrate considerable yields (0.45 mg/g fermenter cell culture) of recombinantly produced protein using *S. cerevisiae*, which is purified to high homogeneity both as opsin (retinal-free) and as functional ChR2 with added retinal. We also develop conditions that enable the growth of ChR2 crystals that scatter X-rays to 6 Å, and identify a molecular replacement solution that suggests that the packing is different from published structures. Consequently, our cost-effective production and purification pipeline opens the way for downstream structural studies of different ChR2 states, which may provide a foundation for further adaptation of this protein for optogenetic applications.

## 1. Introduction

Channelrhodopsins (ChRs) are extraordinary proteins that have revolutionized the field of neurobiology, exploited in transgenic mice to control and map neural circuits [1,2,3]. They function as light-gated ion channels that rapidly depolarize the cell membrane, and have as such attracted enormous interest as optogenetic tools to remotely control muscle and neuron activity in neural cell cultures and living animals [4,5,6]. ChRs have also been explored as novel treatments against severe neurological diseases such as Parkinson’s disease [7], and for restoration of sight in patients [8]. Consequently, these targets are of high biochemical and biomedical relevance.

Channelrhodopsin-1 (ChR1) and Channelrhodopsin-2 (ChR2) from the unicellular algae *Chlamydomonas reinhardtii* were the first identified and characterized light-gated ion channels [4,9]. They primarily serve as sensory photoreceptors to regulate flagellar function that guides the organism to ambient light conditions—a process also known as phototaxis [10]. Additional channelrhodopsins have been identified, such as VChR1 from *Volvox carteri*, CsChR from *Chloromonas subdivisa*, CoChR from *Chloromonas oogama*, and SdChR from *Scherffelia dubia* [11,12]. Because of its high expression level in host cells and activation at lower light intensities, ChR2 is the first choice in optogenetics, and several forms with desirable properties have been engineered [13].

ChRs belong to the large family of microbial rhodopsins. They consist of seven transmembrane helices (TM1–TM7), with a covalently bound retinal cofactor attached to a highly conserved lysine via a protonated so-called “Schiff base” (SB), i.e., linked via the nitrogen atom of the lysine side chain. Light is initially sensed by the bound retinal, triggering a photocycle—a series of conformational changes, some of which are detectable spectrophotometrically, that allow for permeation of a variety of monovalent and divalent cations as well as water-mediated protons in ChRs or, for example, proton pumping in bacteriorhodopsins (bRs) [14,15,16,17,18].

**Figure 1:**
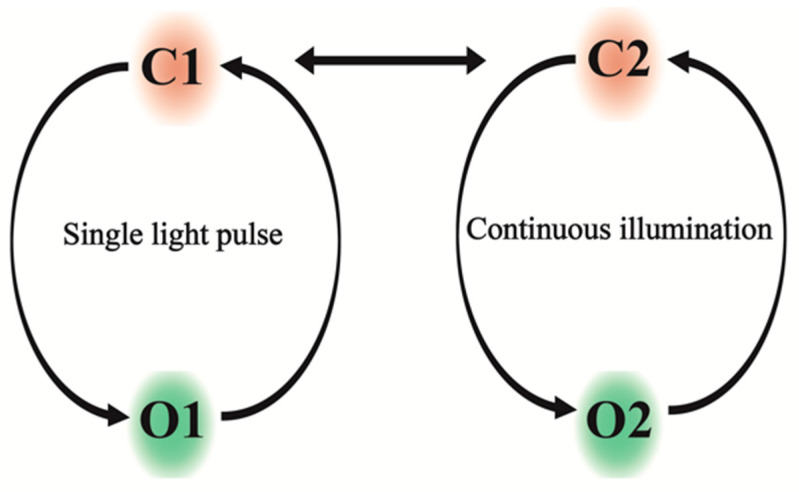
Light-dependent photocycle model of ChR2: To the left and right, the reaction cycles under single light turnover and continuous illumination, respectively, are shown, leading to the conducting open states O1 and O2, respectively. The cycles are interconnected through the closed states.

A variety of methods—such as UV/Vis, FTIR, and Raman spectroscopy, together with mutational studies and high-resolution structural information—have improved our mechanistic understanding of ChRs [19,20,21,22,23]. Two principal states—closed state 1 (C1) and open state 1 (O1)—have been described for ChR2 under single-turnover conditions (Figure 1). Continuous illumination initiates a branched photocycle, with two additional main states: closed state 2 (C2) and open state 2 (O2) [24,25]. The C2 state can revert to the closed state C1 when left in the dark for some time [26]. Compared to the open O1 state of the single-turnover photocycle, the corresponding open O2 state under continuous illumination shows significantly reduced conductance [27]. This dual photocycle provides a foundation for the phenomenon of desensitization, associated with a decay of photocurrents to lower steady-state currents, and light adaptation with smaller peak currents, which cannot be described by a single-turnover reaction cycle [26,27].

Two monomers assemble and form two independent ion conductance pathways, each with four internal cavities separated by three gates [19]. Associated with the central gate and directly linked to the retinal chromophore is the so-called DC pair, formed by residues C128 of TM3 and D156 of TM4, which is of particular functional importance for the channel kinetics [28,29,30]. The C128T mutant, for instance, exhibits a 200-fold extended lifetime [31], and substitution of D156 with alanine or cysteine also results in a dramatically long-lasting open state compared to wild-type ChR2 [29,32]. The current dogma is that light-induced isomerization of retinal leads to rearrangement of the internal hydrogen bonding network, opening the gates for ion passage.

However, to date, no high-resolution structural information of ChR2 in an open or desensitized state is available; hence, details of the ion pathway remain enigmatic, and it is not clear how photoactivation is linked to ion conductivity, or how desensitization and light adaption occur at a molecular level. The latter two processes represent key limitations of ChR2 for exploitation as an optogenetic tool [33]. Therefore, it is also of importance for future neurological and biotechnical applications to understand the molecular basis for light-activated ion conductance in ChR2, thereby permitting downstream rational adaptation of ChRs.

In order to obtain a deeper understanding of the molecular mechanism behind channel opening, structural information on ion-conducting or light-adapted states is critically required. Thus, mutants with an extended lifetime of the conducting state represent attractive forms for such structural studies. However, an existing C128T ChR2 structure revealed an obstructed dark-state conformation [19]. Nevertheless, as substitutions of DC pair residues display different photochemistry, mutant forms of D156 may still be promising to shed further light on the conductance mechanisms [29,30].

Here, we report production and purification procedures of two different D156 ChR2 mutants from *S. cerevisiae*. The folding and stability of the D156C form appears to be independent of retinal, and we describe conditions that yield crystals that diffract to 6 Å and solve the structure using molecular replacement. The packing of this crystal form is different compared to published structures, with two biological dimers in the asymmetric unit.

## 2. Materials and Methods

### 2.1. Construction and Overexpression of Recombinant Plasmids

The cDNAs for two mutants of ChR2—ChR2 (D156C) and ChR2 (D156A)—were provided by Georg Nagel, Julius-von-Sachs-Institut of Biosciences, Julius Maximilian University of Würzburg, Germany. The cDNAs were amplified by PCR with AccuPol DNA polymerase and, together with the cDNA for yEGFP, transformed into the *S. cerevisiae* strain PAP1500 along with BamHI- and HindIII-digested pEMBLyex4 [34] to generate ChR2-GFP-8His expression plasmids via homologous recombination. The sequences of the two plasmid constructs were verified by DNA sequencing (Eurofins, Hamburg Germany). Overproduction of the constructs was performed essentially as described previously [33,35,36,37,38]. After 96 h, the cells were harvested at 3000× *g* for 10 min at 4 °C.

### 2.2. Protein Quantification Estimation by Whole-Cell Fluorescence

A total of 200 μL of the yeast culture was harvested at 1000× *g* for 5 min and resuspended in 200 μL of Milli-Q water. The fluorescence of the GFP-tagged protein constructs was measured on a Spectro microplate fluorometer (Fluoroskan Ascent, Thermo Labsystems, Vantaa, Finland) at 485 nm excitation and 520 nm emission. Milli-Q water was used as a control. The amount of produced protein was calculated with separately purified GFP that was mixed with yeast membranes as a standard, as described elsewhere [35,36].

### 2.3. Bioimaging of Live Yeast Cells

Cellular localization of the C-terminal GFP fusions was detected by live cell bioimaging at 1000× magnification, using a Nikon Eclipse E600 microscope equipped with an Optronics MagnaFire model S99802 camera.

### 2.4. Membrane Preparation

Crude membranes of yeast were prepared by disrupting the cells mechanically with glass beads, as described previously [37]. Briefly, cells were resuspended in lysis buffer containing 20 mM Tris-HCl (pH = 7.5), 500 mM NaCl, and 10% *v/v* glycerol and protease inhibitors (1 mM PMSF and 1 SIGMAFAST protease inhibitor cocktail tablet (Sigma, St. Louis Missouri, MO, USA) at a ratio of 40–50 g wet cell weight for 200 mL of buffer. Cells were lysed through high-speed mixing in the presence of glass beads for 6 × 1 min, with 2 min breaks on ice. The supernatant was collected and the glass beads were washed twice in 50 mL of ice-cold lysis buffer. Unbroken cells and cell debris were removed from the combined supernatants by centrifugation at 5000× *g* for 20 min at 4 °C. The membranes were pelleted via ultracentrifugation at ~142,000× *g* for 3 h at 4 °C. The membranes were resuspended in lysis buffer using a ratio of 1 g of wet weight of membrane per 50 mL of buffer with protease inhibitors, and kept at −80 °C until further usage.

### 2.5. Protein Purification

Crude membranes were thawed on ice and subsequently solubilized in ice-cold solubilization buffer (20 mM Tris-HCl (pH = 7.5), 500 mM NaCl, 10% (*v*/*v*) glycerol, 2 mM β-mercaptoethanol, 1 mM PMSF, and 1 mM retinal supplementation for retinal-bound state purification, without adding 1 mM retinal for non-retinal-bound state purification) supplemented with SIGMAFAST protease inhibitor cocktail by slow stirring for 4 h at 4 °C in the presence of 1% (*w*/*v*) n-dodecyl-β-D-maltopyranoside (DDM, Anatrace, Maumee, OH, USA) and 0.33% (*w*/*v*) cholesteryl hemisuccinate Tris salt (CHS) (Anatrace, Maumee, OH, USA). The solubilized protein was ultracentrifuged at ~142,000× *g* for 30 min at 4 °C and then filtered through a 0.45 μm filter membrane. The solubilized material was loaded onto a 5 mL pre-equilibrated Ni-NTA HisTrap column (Cytiva, München, Germany) and washed and eluted stepwise using imidazole (5 CVs (column volumes) 0 mM imidazole, 10 CVs 60 mM imidazole, and 10 CVs 250 mM imidazole) in purification buffer (20 mM Tris-HCl (pH = 7.5), 500 mM NaCl, 10% (*v*/*v*) glycerol, 2 mM β-mercaptoethanol). The peak fractions were pooled and digested by TEV using a protein-to-TEV ratio of 1:10 (*w*/*w*) during dialysis in buffer (20 mM Tris-HCl (pH = 7.5), 500 mM NaCl, 10% (*v*/*v*) glycerol, 2 mM β-mercaptoethanol, 0.03% (*w*/*v*) DDM, and 0.01% (*w*/*v*) CHS) at 4 °C. After 12–18 h, the digested protein was purified by reverse-affinity purification in order to remove the cleaved GFP-8His tag, His-tagged TEV, uncleaved recombinant protein, and possible contaminants from the initial affinity purification procedure. The sample was concentrated to 10 mg/mL using Vivaspin 50 kDa MWCO concentrators (Vivaproducts, Littleton, MA, USA) and stored at −80 °C. The protein was further purified by size-exclusion chromatography (SEC) using a Superdex increase 200 10/300 GL column (Cytiva) pre-equilibrated in SEC buffer (50 mM sodium citrate (pH = 5.5, 100 mM NaCl, 5% (*v*/*v*) glycerol, 5% (*w*/*v*) sorbitol, 2 mM β-mercaptoethanol, 0.4% (*w*/*v*) n-nonyl-β-D-glucoside (Anatrace, Maumee, OH, USA), and 0.02% (*w*/*v*) CHS). SDS–PAGE was used to assess the purity of samples throughout the purification procedure.

### 2.6. Crystallization and Optimization

The peak fractions from the SEC purification were pooled and concentrated to 5 mg/mL and 10 mg/mL, respectively, using Vivaspin 50 kDa MWCO concentrators, and then crystallized via the hanging-drop vapor-diffusion method. The initial crystals were obtained using 1 μL of protein (5 mg/mL) and 1 μL of precipitant solution of 6% (*v*/*v*) Tacsimate pH = 6.0, 0.1 M MES monohydrate pH = 6.0, and 25% (w/v) polyethylene glycol 4000, from the PEGRx2 commercial screen (Hampton Research). Crystals were optimized, yielding the final crystallization condition, which was used to collect the following data: 41% (*w*/*v*) PEG400, 6% (*w*/*v*) Tacsimate pH = 6.0, and 0.1 M MES pH = 6.0, plus 1% cyclofos-7 and 0.2 M NDSB-195. Crystals were harvested by using Litholoops (Molecular Dimensions, Holland, OH, USA) and flash-cooled in liquid nitrogen.

### 2.7. Data Collection and Analysis

X-ray diffraction data were collected at 110 K at the Swiss Light Source, the Paul Scherrer Institute, Zurich (https://www.psi.ch/sls/, accessed on 3 October 2017), at the X06SA beamline. The collected data were scaled and processed by XDS [38]. The initial phases of the structure were obtained by molecular replacement using PHASER [39] in the Phenix package, with the wild-type ChR2 (PDB-ID:6EID) [19] as a search model.

## 3. Results and Discussion

### 3.1. Production and Localization in S. cerevisiae

**Figure 2:**
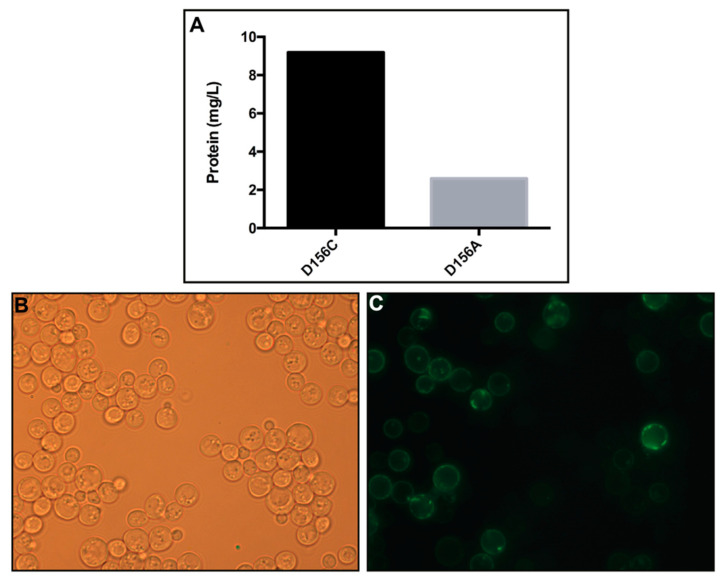
Protein production levels of the ChR2 forms and live cell bioimaging of ChR2 (D156C): (**A**) The measured fluorescence signal in whole cells was converted to protein concentration by applying a GFP standard, as described previously [32]. The scale represents mg of protein per liter of cell culture. (**B**) Differential interference contrast microscopy and (**C**) GFP fluorescence microscopy, showing cells 96 h after induction with galactose.

The lack of structural information on desensitized and open states of ChR2 severely restricts our understanding of the molecular mechanisms underlying light-induced ion passage, light adaptation, and desensitization. With the aim of producing high amounts of ChR2 for comprehensive structural studies on ChR2’s desensitized and open states, the ChR2 mutants D156A and D156C are of particular interest, since they exhibit prolonged channel opening [29,32]. Hitherto, ChR2 has primarily been recombinantly produced in insect cells, worm cells, and *Pichia pastoris* [19,40,41] for structural and functional studies, and through supplementation of retinal in order to obtain stable protein [42]. However, successful post-production addition of retinal has been demonstrated for several microbial rhodopsins synthesized using *E. coli* [43]. In contrast, it has been observed that the limited amount of available all-trans-retinal in *Xenopus* oocytes is insufficient to produce stable ChR2, as the protein is rapidly degraded [44]. When generated in *Drosophila*, it was found that the D156C mutant possessed improved features compared to wild-type ChR2, such as higher expression levels, a prolonged open lifetime, and high photocurrents [32]. In this work, production of ChR2 in *Saccharomyces cerevisiae* was exploited for the first time without the supplementation of retinal, as protein production in yeast is advantageous due to low costs, the fast growth of large cultures, and the ease of genetic manipulation. We applied an inducible production platform especially developed for challenging membrane proteins with a C-terminal GFP fusion [33,35,36], and were able to recover high amounts—up to 9 mg protein/L cell culture of ChR2 (D156C) (Figure 2A). The production levels of the D156A mutant were much lower, and for this reason we focused our work on the D156C mutant. The GFP signal detected in cells suggests that ChR2 was correctly folded, as C-terminal GFP fusions are frequently exploited as reporters of the protein quality [45] (Figure 2B,C).

### 3.2. Purification and Absorption Spectra

**Figure 3:**
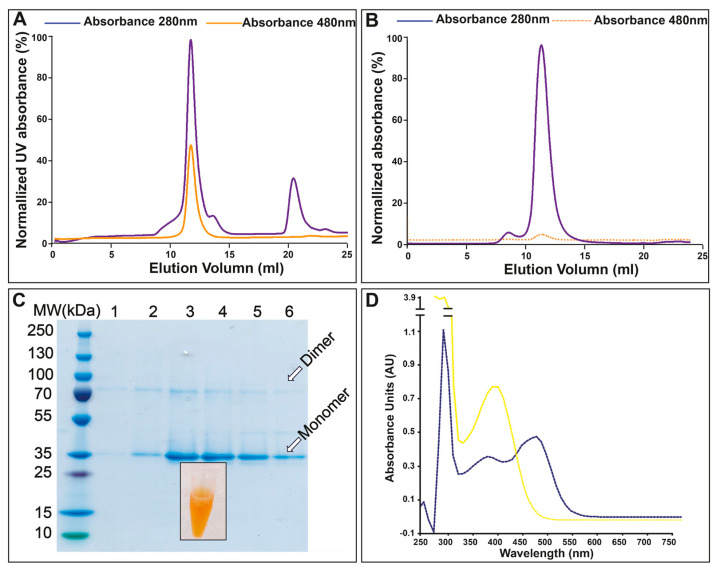
Purification of ChR2 (D156C) and absorption spectra test for free and ChR2–bound retinal: (**A**) Size–exclusion profile of ChR2 (D156C) with retinal supplementation from solubilization of the membranes, and throughout the subsequent purification steps. The blue and orange lines indicate the absorbance at 280 and 480 nm, respectively. (**B**) As in panel A, but without retinal throughout production and purification. (**C**) Coomassie–stained SDS–PAGE of SEC–purified fractions with elution volumes from 11 to 15 mL (lanes 1–6). Insert: an Eppendorf tube containing the purified protein sample. (**D**) The absorption spectrum of free retinal is shown in yellow, with maximum absorption at 380 nm. The absorption spectrum of ChR2 (D156C) supplemented with retinal only during purification, with absorption maxima at 280 nm and 480 nm, is shown in blue. The 280 nm/480 nm ratio for the sample is 2.06, indicative of A well-folded and well-purified ChR2 sample.

It has been reported that the function of the D156C mutant is less dependent on the supplementation of retinal, and this was explained by a higher affinity of the D156C mutant for retinal [32]. In this light, and considering that the cofactor binds covalently to apo-ChR (also known as opsin), we typically only provided retinal (e.g., all-trans, 15-anti) during solubilization, without addition in the pre- or post-purification steps. ChR2 was solubilized from isolated yeast membrane using DDM doped with cholesterol (CHS), and subsequent affinity purification and reverse-affinity purification following removal of the GFP-His tag yielded samples with high purity (Figure 3). Size-exclusion chromatography (SEC) was performed as a final purification step. In line with previous studies, the absorption maximum of retinal-bound D156C was 480 nm; hence, the elution profile of the SEC was monitored at both 280 nm and 480 nm. The obtained SEC profile indicates that the sample is monodisperse, and the position of the peak at 280 nm correlates well with the position of the peak at 480 nm, thus indicating that retinal has bound to the protein, representing a key quality marker of the generated sample as compared to other microbial rhodopsins (Figure 3A) [46,47]. SDS–PAGE analysis of SEC-purified D156C underscores the high purity of the protein, displaying two protein bands that represent monomeric and a dimeric (partially SDS-resistant) forms of the purified protein that have also been reported previously [19] (Figure 3C).

Interestingly, we were also able to produce and purify the D156C mutant in the complete absence of supplemented retinal (throughout the production and purification procedure), obtaining stable protein, as displayed through comparison of the SEC profiles of the D156C form in both the presence and absence of retinal (Figure 3A,B). Subsequent addition of retinal indicated that binding may also be achieved at later stages, as indicated by the absorption shift from 380 nm (free retinal) to 480 nm in the presence of protein—again indicative of well-folded protein (Figure 3D).

### 3.3. Crystallization

**Figure 4:**
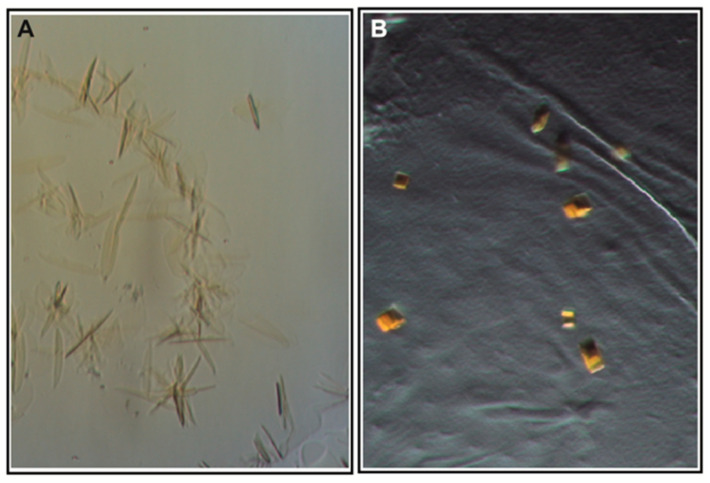
Crystals of ChR2 (D156C): (**A**) Initial crystals grown with reservoir solution containing 6% (*v*/*v*) Tacsimate pH = 6.0, 0.1 M MES monohydrate pH = 6.0, and 25% (*w*/*v*) polyethylene glycol 4000. (**B**) Optimized conditions containing 41% (*v*/*v*) PEG400, 6% (*v*/*v*) Tacsimate pH = 6.0, 0.1 M MES pH = 6.0, 1% cyclofos-7, and 0.2M NDSB-195, yielding cubic crystals.

**Figure 5:**
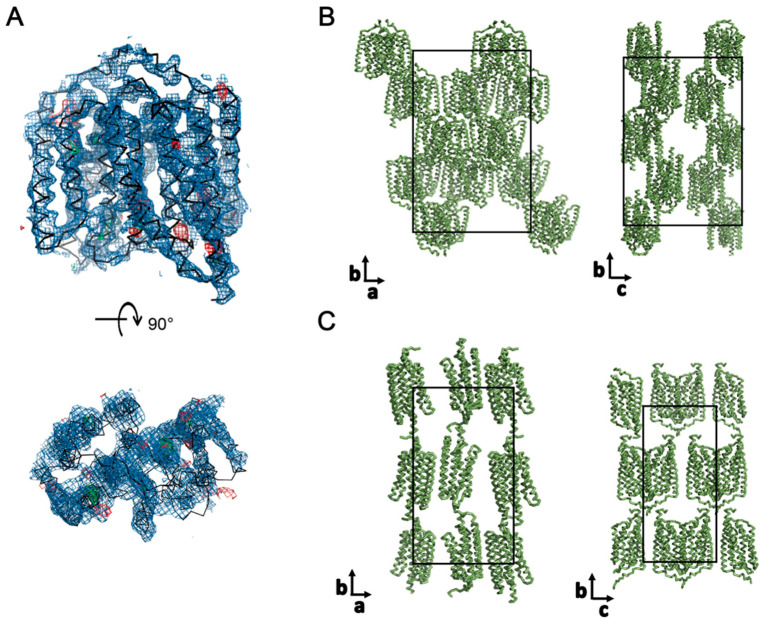
MR solution of the D156C mutant and comparison to the crystal packing of the wild type: (**A**) Molecular replacement solution of the D156C mutant, using the wild-type structure (PDB-ID: 6EID) as a search model. The 2F_O_-F_C_ density map is shown in purple, at σ = 1.0. The F_O_-F_C_ density, with negative values indicated in red and positive values indicated in green, is shown at σ = 3.0. (**B**) The obtained crystal packing of the ChR2 (D156C) crystals. The unit cell is indicated in black. (**C**) Packing of the wild-type ChR2 crystals (PDB ID: 6EID) [19], with the unit cell indicated by a black line.

Crystallization screening with vapor diffusion was performed at 4 and 18 °C, using continuous illumination with blue light, with the aim of obtaining a desensitized or open state of ChR2. Initial clustered crystals (Figure 4A) in 6% *v/v* Tacsimate pH = 6.0, 0.1 M MES monohydrate pH = 6.0, and 25% *w/v* polyethylene glycol 4000 were optimized to separate individual cubic crystals in 41% PEG400, 6% Tacsimate pH = 6.0 and 0.1M MES pH = 6.0, cyclofos-7, and NDSB-195 (Figure 4B). These crystals diffracted to 6 Å and the space group was determined to be C222, with unit cell parameters a = 117.2 Å, b = 179.9 Å, and c = 125.0 Å (Table 1). A molecular replacement (MR) solution was identified using Phaser [35] by performing the search with one biological dimer of the wild-type structure (PDB-ID: 6EID) [19] (Figure 5A), yielding a translation function Z-score of 5.7 and a log-likelihood gain of 73.8 [39]. Notably, a comparable solution was also found by providing only one monomer of the wild-type structure as the search model, strengthening the correctness of the molecular replacement solution.

The crystals pack as type I crystals, and two biological dimers are found in the asymmetric unit (Figure 5B) in contrast to the wild type and the C128T structure, where the asymmetric unit consists of two inversely orientated protomers (Figure 5C) [19]. The different packing and crystal contacts in the D156C mutant may indicate a distinct conformation compared to previously published structures, but optimization of the crystals to yield higher resolution data will be required in order to refine and confidently interpret the structural model in detail.

## 4. Conclusions

Our established procedure for the production and purification of high amounts of pure and stable ChR2 (D156C) will be beneficial for downstream crystal optimization efforts. Considering that spectroscopic, structural, and computational studies hint that the conformational changes in ChRs are substantial, densely packed crystals such as those reported from LCP experiments may not be amenable for light-triggered structural studies. In contrast, the alternative packing obtained here using vapor diffusion may be more suitable for such studies, following further optimization. However, it remains possible that complementary crystal forms—e.g., those grown in detergent, in meso, or in the presence of high concentrations of lipids and detergent—may be applicable as well [48,49,50]. Nevertheless, the isolation method and the particular ChR2 mutant form likely pave the way for the structural determination of an open-state structure, but such an ambition may also require time- and light-resolved data collection strategies, as previously successfully applied to obtain high-resolution structures of, for example, bacteriorhodopsin [51]. Nevertheless, the D156C mutant is especially promising for these purposes, due to its prolonged open lifetime compared to the wild-type ChR2 [32], as well as its high production levels and stability, as reported here. Thus, the ChR2 (D156C) mutant deserves more attention so as to obtain further structural information to shed more light on the overall reaction cycle, and thereby to enable modulation of ChR2 for optogenetic applications.

## Figures and Tables

**Table 1:** Summary of data collection statistics. Values in parentheses are for the highest resolution shell.

Space Group	C222
a, b, c, Å	117.095 179.928 126.471
α, β, γ, °	90.00 90.00 90.00
Resolution, Å	50–5.0 (5.3–5.0)
R_merge_ %	30.1 (337.5)
I/σ	2.7 (0.5)
Completeness, %	99.4 (98.4)
CC(1/2)	100.0 (79.4)

## Data Availability

Not applicable.

## References

[1] Arenkiel B.R., Peca J., Davison I.G., Feliciano C., Augustine G.J., Ehlers M.D., Feng G. (2007). In Vivo Light-Induced Activation of Neural Circuitry in Transgenic Mice Expressing Channelrhodopsin-2. Neuron.

[2] Wang H., Peca J., Matsuzaki M., Matsuzaki K., Noguchi J., Qiu L., Wang D., Zhang F., Boyden E., Deisseroth K. (2007). High-speed mapping of synaptic connectivity using photostimulation in Channelrhodopsin-2 transgenic mice. Proc. Natl. Acad. Sci. USA.

[3] Petreanu L., Huber D., Sobczyk A., Svoboda K. (2007). Channelrhodopsin-2-assisted circuit mapping of long-range callosal projections. Nat. Neurosci..

[4] Kateriya S., Berthold P., Bamberg E., Huhn W., Nagel G., Hegemann P., Adeishvili N., Szellas T., Ollig D. (2003). Channelrhodopsin-2, a directly light-gated cation-selective membrane channel. Proc. Natl. Acad. Sci. USA.

[5] Ernst O.P., Hegemann P., Tsunoda S.P., Gradmann D., Mages W., Berthold P. (2008). Channelrhodopsin-1 Initiates Phototaxis and Photophobic Responses in Chlamydomonas by Immediate Light-Induced Depolarization. Plant Cell Online.

[6] Josselyn S.A. (2018). The past, present and future of light-gated ion channels and optogenetics. Elife.

[7] Steinbeck J.A., Choi S.J., Mrejeru A., Ganat Y., Deisseroth K., Sulzer D., Mosharov E.V., Studer L. (2015). Optogenetics enables functional analysis of human embryonic stem cell-derived grafts in a Parkinson’s disease model. Nat. Biotechnol..

[8] Scholl H.P.N., Strauss R.W., Singh M.S., Dalkara D., Roska B., Picaud S., Sahel J.-A. (2016). Emerging therapies for inherited retinal degeneration. Sci. Transl. Med..

[9] Nagel G., Ollig D., Fuhrmann M., Kateriya S., Musti A.M., Bamberg E., Hegemann P. (2002). Channelrhodopsin-1: A light-gated proton channel in green algae. Science.

[10] Harz H., Hegemann P. (1991). Rhodopsin-regulated calcium currents in Chlamydomonas. Nature.

[11] Zhang F., Prigge M., Beyrière F., Tsunoda S.P., Mattis J., Yizhar O., Hegemann P., Deisseroth K. (2008). Red-shifted optogenetic excitation: A tool for fast neural control derived from Volvox carteri. Nat. Neurosci..

[12] Klapoetke N.C., Murata Y., Kim S.S., Pulver S.R., Birdsey-Benson A., Cho Y.K., Morimoto T.K., Chuong A.S., Carpenter E.J., Tian Z. (2014). Independent optical excitation of distinct neural populations. Nat. Methods.

[13] Lin J.Y. (2010). A user’s guide to channelrhodopsin variants: Features, limitations and future developments. Exp. Physiol..

[14] Hegemann P., Gärtner W., Uhl R. (1991). All-trans retinal constitutes the functional chromophore in Chlamydomonas rhodopsin. Biophys. J..

[15] Ernst O.P., Sánchez Murcia P.A., Daldrop P., Tsunoda S.P., Kateriya S., Hegemann P. (2008). Photoactivation of channelrhodopsin. J. Biol. Chem..

[16] Lawson M.A., Zacks D.N., Derguini F., Nakanishi K., Spudich J.L. (1991). Retinal analog restoration of photophobic responses in a blind Chiamydomonas reinhardtii mutant. Biophys. J..

[17] Bamann C., Kirsch T., Nagel G., Bamberg E. (2008). Spectral Characteristics of the Photocycle of Channelrhodopsin-2 and Its Implication for Channel Function. J. Mol. Biol..

[18] Kandori H., Yamazaki Y., Sasaki J., Maeda A., Needleman R., Lanyi J.K. (1995). Water-Mediated Proton Transfer in Proteins: An FTIR Study of Bacteriorhodopsin. J. Am. Chem. Soc..

[19] Volkov O., Kovalev K., Polovinkin V., Borshchevskiy V., Bamann C., Astashkin R., Marin E., Popov A., Balandin T., Willbold D. (2017). Structural insights into ion conduction by channelrhodopsin 2. Science.

[20] Lórenz-Fonfría V.A., Heberle J. (2014). Proton Transfer and Protein Conformation Dynamics in Photosensitive Proteins by Time-resolved Step-scan Fourier-transform Infrared Spectroscopy. J. Vis. Exp..

[21] Muders V., Kerruth S., Lórenz-Fonfría V.A., Bamann C., Heberle J., Schlesinger R. (2014). Resonance Raman and FTIR spectroscopic characterization of the closed and open states of channelrhodopsin-1. FEBS Lett..

[22] Ritter E., Puskar L., Bartl F.J., Aziz E.F., Hegemann P., Schade U. (2015). Time-resolved infrared spectroscopic techniques as applied to channelrhodopsin. Front. Mol. Biosci..

[23] Ito S., Kato H.E., Taniguchi R., Iwata T., Nureki O., Kandori H. (2014). Water-containing hydrogen-bonding network in the active center of channelrhodopsin. J. Am. Chem. Soc..

[24] Hegemann P., Ehlenbeck S., Gradmann D. (2005). Multiple photocycles of channelrhodopsin. Biophys. J..

[25] Nikolic K., Grossman N., Grubb M.S., Burrone J., Toumazou C., Degenaar P. (2009). Photocycles of channelrhodopsin-2. Photochem. Photobiol..

[26] Saita M., Pranga-Sellnau F., Resler T., Schlesinger R., Heberle J., Lorenz-Fonfria V.A. (2018). Photoexcitation of the P4480State Induces a Secondary Photocycle That Potentially Desensitizes Channelrhodopsin-2. J. Am. Chem. Soc..

[27] Kuhne J., Vierock J., Alexander S., Dreier M., Wietek J. (2018). Title : A unifying photocycle model for light adaptation and temporal evolution of cation conductance in Channelrhodopsin-2. Proc. Natl. Acad. Sci. USA.

[28] Nack M., Radu I., Gossing M., Bamann C., Bamberg E., Von Mollard G.F., Heberle J. (2010). The DC gate in channelrhodopsin-2: Crucial hydrogen bonding interaction between C128 and D156. Photochem. Photobiol. Sci..

[29] Bamann C., Gueta R., Kleinlogel S., Nagel G., Bamberg E. (2010). Structural guidance of the photocycle of channelrhodopsin-2 by an interhelical hydrogen bond. Biochemistry.

[30] Lorenz-Fonfria V.A., Resler T., Krause N., Nack M., Gossing M., Fischer von Mollard G., Bamann C., Bamberg E., Schlesinger R., Heberle J. (2013). Transient protonation changes in channelrhodopsin-2 and their relevance to channel gating. Proc. Natl. Acad. Sci. USA.

[31] Stehfest K., Ritter E., Berndt A., Bartl F., Hegemann P. (2010). The branched photocycle of the slow-cycling channelrhodopsin-2 mutant C128T. J. Mol. Biol..

[32] Dawydow A., Gueta R., Ljaschenko D., Ullrich S., Hermann M., Ehmann N., Gao S., Fiala A., Langenhan T., Nagel G. (2014). Channelrhodopsin-2-XXL, a powerful optogenetic tool for low-light applications. Proc. Natl. Acad. Sci. USA.

[33] Fenno L., Yizhar O., Deisseroth K. (2011). The Development and Application of Optogenetics. Annu. Rev. Neurosci..

[34] Felici F., Cesareni G. (1987). Structure of the Saccharomyces cerevisiae gene encoding tRNAIle (IAU). Nucleic Acids Res..

[35] Bomholt J., Helix-Nielsen C., Scharff-Poulsen P., Pedersen P.A. (2013). Recombinant production of human Aquaporin-1 to an exceptional high membrane density in Saccharomyces cerevisiae. PLoS ONE.

[36] Scharff-Poulsen P., Pedersen P.A. (2013). Saccharomyces cerevisiae-Based Platform for Rapid Production and Evaluation of Eukaryotic Nutrient Transporters and Transceptors for Biochemical Studies and Crystallography. PLoS ONE.

[37] Jørgensen J.R., Pedersen P.A. (2001). Role of phylogenetically conserved amino acids in folding of Na,K-ATPase. Biochemistry.

[38] Kabsch W. (2010). Xds. Acta Crystallogr. Sect. D Biol. Crystallogr..

[39] McCoy A.J., Grosse-Kunstleve R.W., Adams P.D., Winn M.D., Storoni L.C., Read R.J. (2007). Phaser crystallographic software. J. Appl. Crystallogr..

[40] Kato H.E., Zhang F., Yizhar O., Ramakrishnan C., Nishizawa T., Hirata K., Ito J., Aita Y., Tsukazaki T., Hayashi S. (2012). Crystal structure of the channelrhodopsin light-gated cation channel. Nature.

[41] Mazza F. (2014). Expression and Purification of Channelrhodopsin-2 in Pichia Pastoris: Generation of Blue-Shifted and Redshifted Optogenetic Variants. Ph.D. Thesis.

[42] Ullrich S., Gueta R., Nagel G. (2013). Degradation of channelopsin-2 in the absence of retinal and degradation resistance incertain mutants. Biol. Chem..

[43] Overproduction P., Hunter C.N. (2020). Proteorhodopsin Overproduction Enhances the Long-Term Viability of Escherichia coli. Appl. Environ. Microbiol..

[44] Seki T., Fujishita S., Ito M., Matsuoka N., Tsukida K. (1987). Retinoid composition in the compound eyes of insects. Exp. Biol..

[45] Waldo G.S., Arnold F.H., Georgiou G. (2003). Improving Protein Folding Efficiency by Directed Evolution Using the GFP Folding Reporter. Directed Enzyme Evolution: Screening and Selection Methods.

[46] Gourdon P., Alfredsson A., Pedersen A., Malmerberg E., Nyblom M., Widell M., Berntsson R., Pinhassi J., Braiman M., Hansson Ö. (2008). Optimized in vitro and in vivo expression of proteorhodopsin: A seven-transmembrane proton pump. Protein Expr. Purif..

[47] Krause N. (2016). Structural Rearrangements upon Opening of Channelrhodopsin-2. Ph.D. Thesis.

[48] Bill R.M., Henderson P.J.F., Iwata S., Kunji E.R.S., Michel H., Neutze R., Newstead S., Poolman B., Tate C.G., Vogel H. (2011). Overcoming barriers to membrane protein structure determination. Nat. Biotechnol..

[49] Gourdon P., Andersen J.L., Hein K.L., Bublitz M., Pedersen B.P., Liu X.Y., Yatime L., Nyblom M., Nielsen T.T., Olesen C. (2011). HiLiDe-systematic approach to membrane protein crystallization in lipid and detergent. Cryst. Growth Des..

[50] Caffrey M. (2015). A comprehensive review of the lipid cubic phase or in meso method for crystallizing membrane and soluble proteins and complexes. Acta Crystallogr. Sect. FStructural Biol. Commun..

[51] Royant A., Edman K., Ursby T., Pebay-Peyroula E., Landau E.M., Neutze R. (2000). Helix deformation is coupled to vectorial proton transport in the photocycle of bacteriorhodopsin. Nature.

